# The glycan‐dependent ERAD machinery degrades topologically diverse misfolded proteins

**DOI:** 10.1111/tpj.13851

**Published:** 2018-03-14

**Authors:** Yun‐Ji Shin, Ulrike Vavra, Christiane Veit, Richard Strasser

**Affiliations:** ^1^ Department of Applied Genetics and Cell Biology University of Natural Resources and Life Sciences Muthgasse 18 Vienna A‐1190 Austria

**Keywords:** endoplasmic reticulum, glycosylation, integral membrane protein, protein folding, quality control

## Abstract

Many soluble and integral membrane proteins fold in the endoplasmic reticulum (ER) with the help of chaperones and folding factors. Despite these efforts, protein folding is intrinsically error prone and amino acid changes, alterations in post‐translational modifications or cellular stress can cause protein misfolding. Folding‐defective non‐native proteins are cleared from the ER and typically undergo ER‐associated degradation (ERAD). Here, we investigated whether different misfolded glycoproteins require the same set of ERAD factors and are directed to HRD1 complex‐mediated degradation in plants. We generated a series of glycoprotein ERAD substrates harboring a misfolded domain from Arabidopsis STRUBBELIG or the BRASSINOSTEROID INSENSITVE 1 receptor fused to different membrane anchoring regions. We show that single pass and multispanning ERAD substrates are subjected to glycan‐dependent degradation by the HRD1 complex. However, the presence of a powerful ER exit signal in the multispanning ERAD substrates causes competition with ER quality control and targeting of misfolded glycoproteins to the vacuole. Our results demonstrate that the same machinery is used for degradation of topologically different misfolded glycoproteins in the ER of plants.

## Significance Statement

Protein quality control mechanisms in the endoplasmic reticulum ensure that only correctly folded proteins are delivered to other compartments of the secretory pathway, while non‐native (potentially deleterious) proteins are degraded. This study reveals that a distinct ER‐associated degradation (ERAD) pathway is involved in clearance of different types of aberrant glycoproteins.

## Introduction

In all eukaryotes, a dedicated quality control system in the endoplasmic reticulum (ER) maintains protein homeostasis and sends aberrant proteins for degradation to prevent their accumulation and the secretion of potentially harmful proteins. Protein folding is inherently error prone and affected by factors such as genetic mutations or post‐translational modifications (Balchin *et al*., 2016). While ER quality control mechanisms are aiming to retain incompletely folded proteins in the ER for further folding reactions, such folding‐defective proteins frequently carry functional signals for ER export and targeting to other cellular compartments like the Golgi apparatus or the plasma membrane. Consequently, ER quality control competes with ER export processes (Vashist *et al*., 2001; Sekijima *et al*., 2005; Kincaid and Cooper, 2007; Kawaguchi *et al*., 2010). Approximately one‐third of all proteins from eukaryotes are synthesized at the ER membrane and targeted to the secretory pathway. Since these proteins are very heterogeneous and comprise soluble as well as different types of membrane‐anchored and glycosylated/non‐glycosylated proteins, ER quality control processes have to be quite flexible to handle this diverse range of substrates (Roversi *et al*., 2017). Yet, it is poorly understood how the ER quality control machinery interacts with different clients and decides their fate.

The ER‐associated degradation (ERAD) pathway is a highly conserved process that orchestrates the degradation of aberrant or unassembled proteins during ER quality control. For budding yeast and metazoans, the classic ERAD pathway involves recognition of the aberrant protein, delivery to a specific membrane‐embedded protein complex that contains an E3 ubiquitin ligase, translocation across the membrane bilayer into the cytosol, ubiquitination and eventually proteasomal degradation (Vembar and Brodsky, 2008). In the current view, proteins with a misfolded luminal domain are degraded by the ER membrane resident E3 ubiquitin ligase HRD1. The Arabidopsis genome encodes two HRD1 homologs (HRD1A and HRD1B) with redundant functions (Su *et al*., 2011). The multi‐pass membrane proteins HRD1A/HRD1B are required for the degradation of a misfolded variant of the BRASSINOSTEROID INSENSITVE 1 receptor (BRI1) (Su *et al*., 2011) and involved in tuning of other ERAD components (Chen *et al*., 2016). The plant HRD1 complex participates in abiotic and ER stress responses in Arabidopsis (Liu *et al*., 2011; Hüttner and Strasser, 2012; Hüttner *et al*., 2012; Su *et al*., 2012; Chen *et al*., 2016; Van Hoewyk, 2016) and is implicated in the formation of protein bodies in the rice endosperm (Ohta and Takaiwa, 2015). In addition to HRD1, plants possess other ER membrane located E3 ubiquitin ligases such as DOA10 involved in the clearance of endogenous or overexpressed misfolded proteins (Doblas *et al*., 2013; Pollier *et al*., 2013; Chen *et al*., 2016). Together with DOA10, HRD1 negatively regulates, for example, thermotolerance in Arabidopsis (Li *et al*., 2017).

BRI1 is a heavily glycosylated protein and variants with misfolded luminal domains (BRI1‐5, BRI1‐9) are subjected to glycan‐dependent ERAD (Jin *et al*., 2007; Hong *et al*., 2008). This pathway can be efficiently blocked with kifunensine, a specific class I α‐mannosidase inhibitor (Elbein *et al*., 1990). In the presence of kifunensine the trimming of α1,2‐linked mannose residues from *N*‐glycans is blocked which prevents the formation of a conserved glycan determinant required for ERAD of aberrant glycoproteins (Clerc *et al*., 2009; Hong *et al*., 2012). Consistent with these findings, it has been shown that the deficiency of MNS4 and MNS5, two class I α‐mannosidases from Arabidopsis with a largely redundant function, prevents the degradation of misfolded BRI1 and rescues the associated growth defects of *bri1‐5* and *bri1‐9* mutants (Hüttner *et al*., 2014b). Other recently identified components of the ERAD pathway that act in the recognition and targeting steps in plants are the luminal lectin‐like protein OS9 and the type I membrane protein SEL1L (also called HRD3) (Liu *et al*., 2011; Su *et al*., 2011, 2012; Hüttner *et al*., 2012; Ohta and Takaiwa, 2015). Together these proteins form the OS9‐SEL1L‐HRD1 ERAD complex (also called HRD1 complex) that is highly conserved from yeast to mammals and plants (Christianson and Ye, 2014; Hüttner *et al*., 2014b; Liu and Li, 2014).

In *Saccharomyces cerevisiae*, membrane‐tethered folding‐defective proteins appear degraded by a similar pathway and kinetics as the same protein lacking a membrane anchor (Taxis *et al*., 2003; Vashist and Ng, 2004). Disposal of glycoprotein ERAD substrates in mammalian cells is, however, more complex as the presence or absence of a membrane anchor targets misfolded glycoproteins to different disposal pathways (Bernasconi *et al*., 2010). The presence of single or multi‐pass transmembrane domains on misfolded proteins is energetically less favoured and imposes a challenge for their degradation which could explain the targeting to an alternative pathway (Baldridge and Rapoport, 2016). Recently, an ER‐resident guardian protein complex was identified in yeast that protects folding intermediates from degradation and is specific for soluble proteins (Zhang *et al*., 2017). Collectively, these studies indicate that targeting and/or membrane anchoring regions can affect the clearance of misfolded proteins and compete with cellular processes like glycan‐dependent ERAD. While these pathways and the underlying processes have been addressed in mammalian cells and budding yeast, the requirement for glycan‐dependent ERAD of different folding‐defective glycoproteins is largely unknown in plants (Liu and Li, 2014).

In a previous study we have shown that a misfolded variant of the extracellular domain from Arabidopsis STRUBBELIG (SUBEX‐C57Y) is a canonical ERAD substrate that is degraded in a glycan‐dependent manner in plants (Hüttner *et al*., 2014a). STRUBBELIG is a leucine‐rich repeat receptor‐like kinase that is crucial for tissue morphogenesis of different plant organs (Vaddepalli *et al*., 2011). The luminal glycoprotein ERAD substrate SP‐SUBEX‐C57Y‐GFP contains a signal peptide (SP) for entering the secretory pathway, three *N*‐glycans and a single amino acid substitution of a conserved cysteine in the *N*‐terminal capping domain of STRUBBELIG. SP‐SUBEX‐C57Y‐GFP is retained in the ER and its degradation requires mannose trimming mediated by MNS4 or MNS5 and components of the OS9‐SEL1L‐HRD1 ERAD pathway (Hüttner *et al*., 2014a). Here, we generated a panel of chimeric membrane‐anchored variants of the misfolded SUBEX‐C57Y glycoprotein ERAD substrate and systematically analysed their stability and subcellular fate to resolve the interplay between membrane anchoring, functional ER export signals and ERAD in plants. Our data reveal that tethering of a folding‐defective ERAD substrate with different membrane anchors does not obstruct ERAD mediated by the glycan‐dependent HRD1 pathway.

## Results

### Membrane anchoring with a cytoplasmic‐transmembrane and stem region from an ER‐resident type II membrane protein does not interfere with glycan‐dependent ERAD

**Figure 1:**
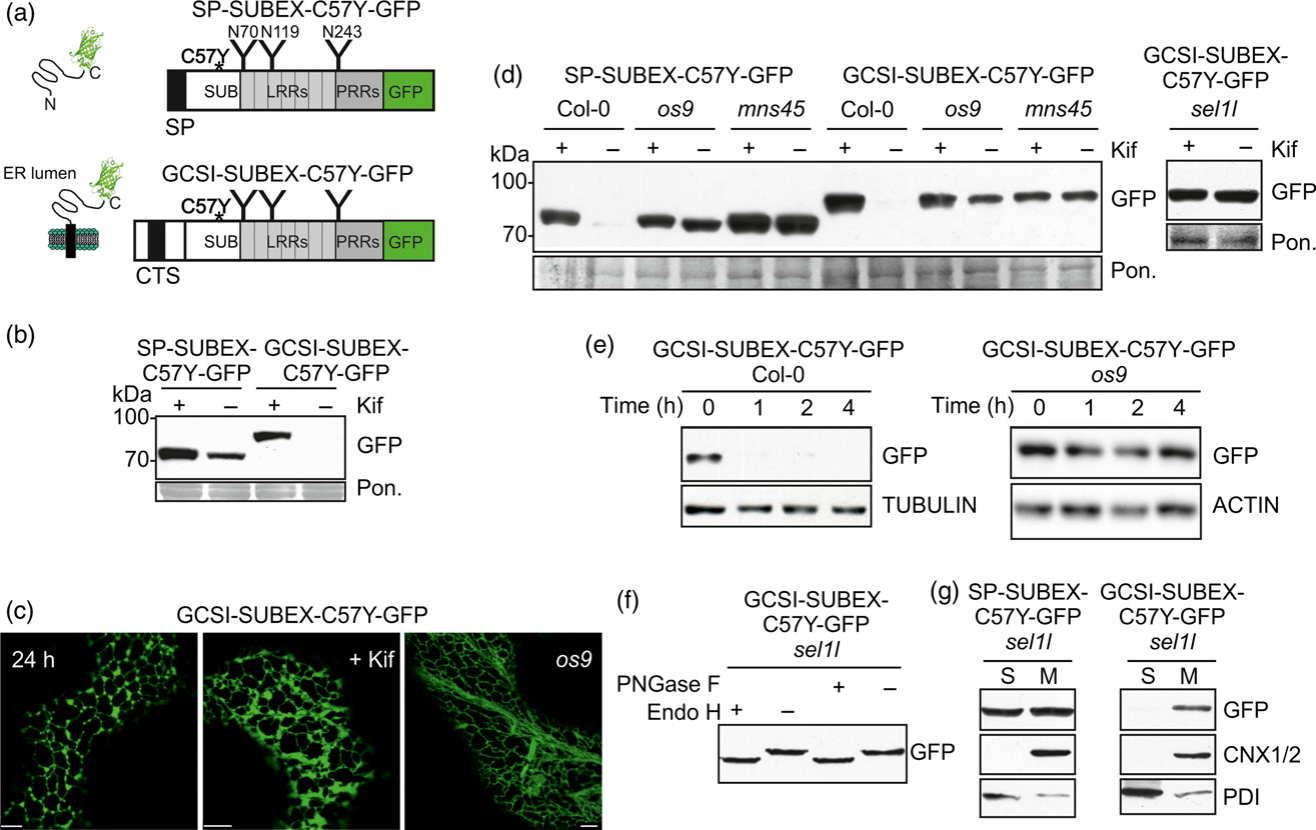
A misfolded ER‐retained type II membrane protein is subjected to glycan‐dependent ERAD.(a) Schematic illustration of SP‐SUBEX‐C57Y‐GFP and GCSI‐SUBEX‐C57Y‐GFP variants. The asterisk denotes the position of the amino acid change (C57Y) leading to misfolding; ‘Y’ indicates the position of the *N*‐glycosylation sites; CTS, cytoplasmic‐transmembrane domain‐stem region; GFP, green fluorescent protein; LRRs, leucine‐rich repeats; PRRs, proline‐rich repeats; SP, signal peptide; SUB, SUB‐domain from STRUBBELIG.(b) Immunoblot analysis of GCSI‐SUBEX‐C57Y‐GFP transiently expressed in *N*. *benthamiana* leaves in the presence or absence of 20 μm kifunensine (Kif). Ponceau S staining (Pon.) is shown as a loading control. Samples were harvested 48 h after infiltration.(c) Confocal images of transiently (24 h post infiltration in *N*. *benthamiana* leaves) or stable expressed GCSI‐SUBEX‐C57Y‐GFP (in leaves of 4–5‐week‐old Arabidopsis *os9* plants). Scale bars = 5 μm.(d) Immunoblot analysis of stable expressed SP‐SUBEX‐C57Y‐GFP and GCSI‐SUBEX‐C57Y‐GFP in leaves from 4–5‐week‐old Arabidopsis Col‐0, *os9*,* mns45* and *sel1l*.(e) Degradation of GCSI‐SUBEX‐C57Y‐GFP in Col‐0 and *os9* in the presence of 100 μg/ml cycloheximide (CHX). The experiment was done three times with similar results.(f) Endo H and PNGase F digestion of GCSI‐SUBEX‐C57Y‐GFP extracted from 4–5‐week‐old Arabidopsis *sel1l*.(g) Detection of SP‐SUBEX‐C57Y‐GFP and GCSI‐SUBEX‐C57Y‐GFP in soluble (S) and membrane fractions (M) of 12‐day‐old *sel1l* seedlings. The membrane fraction is detected with an antibody against CALNEXIN 1/2 (CNX1/2) and protein disulfide isomerase (PDI) serves as a control for the soluble fraction. [Colour figure can be viewed at http://wileyonlinelibrary.com].

To investigate the effect of *N*‐terminal membrane anchoring regions on ERAD we generated chimeric protein variants where the same misfolded glycoprotein was linked to a protein region for membrane anchoring. For this purpose, the signal peptide sequence of the luminal ERAD substrate SP‐SUBEX‐C57Y was replaced by the cytoplasmic‐transmembrane and stem (CTS) region from the ER‐resident *N*‐glycan processing enzyme Arabidopsis α‐glucosidase I (GCSI), from the *cis*‐Golgi‐resident α‐mannosidase I (MNS1) or from the *trans*‐Golgi‐resident rat α2,6‐sialyltransferase (ST). These CTS regions are well characterized in plants and have been used in several studies to target proteins to the ER (GCSI) and Golgi apparatus (MNS1 and ST) (Boevink *et al*., 1998; Saint‐Jore‐Dupas *et al*., 2006; Liebminger *et al*., 2009; Schoberer *et al*., 2013). First, we analysed whether the ER‐resident GCSI‐SUBEX‐C57Y‐GFP is directed to a glycan‐dependent ERAD pathway (Figure 1a). GCSI‐SUBEX‐C57Y‐GFP was transiently expressed in *N*. *benthamiana* leaves in the presence or absence of the ERAD inhibitor kifunensine which blocks mannose trimming from *N*‐glycans and leads to the accumulation of misfolded glycoproteins (Hüttner *et al*., 2014a). Similar to the luminal SP‐SUBEX‐C57Y‐GFP, GCSI‐SUBEX‐C57Y‐GFP protein levels were drastically increased in the presence of the ERAD inhibitor (Figure 1b). Confocal microscopy of transiently expressed GCSI‐SUBEX‐C57Y‐GFP revealed a typical ER type localization (Figure 1c). To test whether GCSI‐SUBEX‐C57Y‐GFP degradation is dependent on the known ERAD factors OS9, SEL1L and MNS4/MNS5 (Liu *et al*., 2011; Su *et al*., 2011, 2012; Hüttner *et al*., 2012, 2014b) we generated transgenic Arabidopsis in the respective genetic knockouts and analysed the protein levels by immunoblotting. In Col‐0 wild‐type plants, GCSI‐SUBEX‐C57Y‐GFP behaved like the soluble ERAD substrate SP‐SUBEX‐C57Y‐GFP and was only detectable in the presence of kifunensine (Figure 1d). By contrast, clear accumulation was observed in *os9* and *sel1l* single mutants as well as in the *mns45* double mutant. The accumulation of the misfolded protein was not further increased by kifunensine indicating that GCSI‐SUBEX‐C57Y‐GFP is subjected to ERAD and its glycan‐dependent degradation is completely blocked in these mutant lines.

To obtain data on the degradation kinetics, we inhibited protein synthesis in seedlings with cycloheximide (CHX) and analysed the decrease in protein abundance at different times. Immunoblot analysis of CHX treated seedlings revealed the rapid clearance of the misfolded protein in wild‐type seedlings and substantial stabilization in the *os9* mutant with the blocked ERAD pathway (Figure 1e). Confocal microscopy of *os9* seedlings showed that GCSI‐SUBEX‐C57Y‐GFP is primarily present in the ER when ERAD is blocked (Figure 1c). Retention in the ER results in the presence of incompletely processed oligomannosidic *N*‐glycans on glycoproteins, while targeting to the Golgi leads to the formation of processed complex *N*‐glycans. These two possibilities can be distinguished by endoglycosidase H (Endo H) digestion which cleaves off oligomannosidic *N*‐glycans but not Golgi‐processed complex ones. Endo H digestion and immunoblotting of protein extracts from *sel1l* expressing GCSI‐SUBEX‐C57Y‐GFP resulted in a clear shift in mobility (Figure 1f). The same shift was observed when protein extracts were digested with PNGase F which removes oligomannosidic and complex *N*‐glycans lacking core α1,3‐fucose. As the endoglycosidase digestions showed the presence of ER‐typical oligomannosidic *N*‐glycans we concluded that the GCSI‐SUBEX‐C57Y‐GFP protein is not in contact with Golgi‐resident *N*‐glycan processing enzymes. Next, we analysed soluble and membrane fractions from seedlings for the presence of the ERAD substrate. In the *sel1l* genetic background GCSI‐SUBEX‐C57Y‐GFP was mainly associated with the membrane fraction, while SP‐SUBEX‐C57Y‐GFP was found in the soluble and membrane fraction (Figure 1g). Together, these results show that a membrane‐anchored ER‐resident glycoprotein ERAD substrate is subjected to degradation by the MNS4/MNS5‐OS9‐SEL1L ERAD complex in plants.

### A functional ER exit and Golgi retention signal from a type II membrane protein does not affect glycan‐dependent ERAD in plants

**Figure 2:**
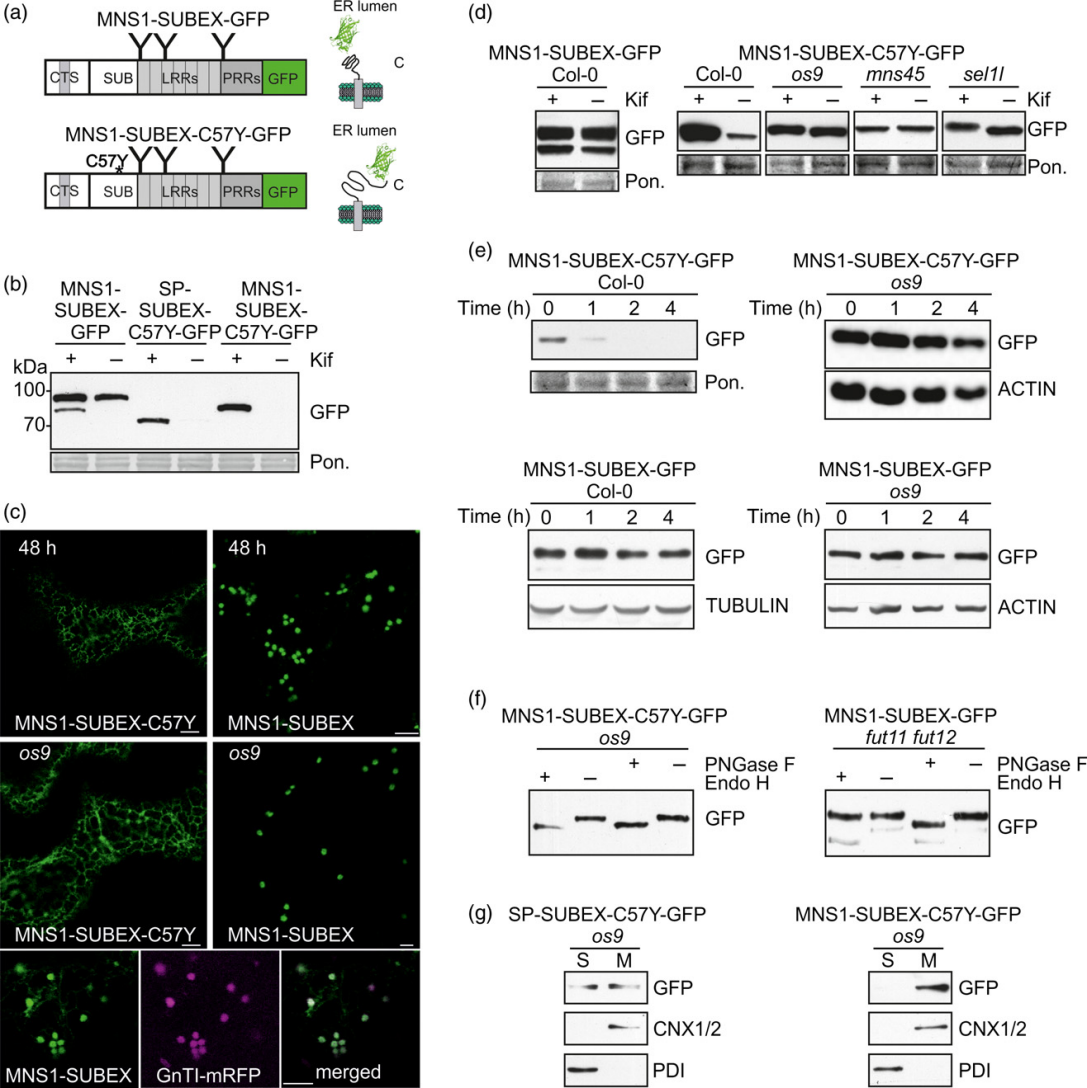
The ERAD pathway is dominant over Golgi targeting/retention signals from type II membrane proteins.(a) Schematic illustration of MNS1‐SUBEX‐GFP and MNS1‐SUBEX‐C57Y‐GFP variants.(b) Immunoblot of MNS1‐SUBEX‐GFP and MNS1‐SUBEX‐C57Y‐GFP transiently expressed in *N*. *benthamiana* leaves (48 h post infiltration) in the presence or absence of 20 μm kifunensine (Kif).(c) Confocal images of transiently (48 h post infiltration in *N*. *benthamiana* leaves) and stable (in leaves of 4–5‐week‐old Arabidopsis *os9*) expressed MNS1‐SUBEX‐C57Y‐GFP and MNS1‐SUBEX‐GFP. Analysis of co‐localization was done by co‐expression of MNS1‐SUBEX‐GFP and the Golgi marker GnTI‐mRFP in *N*. *benthamiana* leaves. Scale bars = 5 μm.(d) Immunoblot analysis of protein extracts from 4–5‐week‐old Arabidopsis expressing MNS1‐SUBEX‐GFP and MNS1‐SUBEX‐C57Y‐GFP.(e) Degradation of MNS1‐SUBEX‐C57Y‐GFP and MNS1‐SUBEX‐GFP in the presence of 100 μg ml^−1^ cycloheximide (CHX). The experiment was done three times with similar results.(f) Endo H and PNGase F digestion of MNS1‐SUBEX‐GFP and MNS1‐SUBEX‐C57Y‐GFP.(g) Detection of SP‐SUBEX‐C57Y‐GFP and MNS1‐SUBEX‐C57Y‐GFP in soluble (S) and membrane fractions (M) of 12‐day‐old *os9* seedlings. [Colour figure can be viewed at http://wileyonlinelibrary.com].

We next asked whether a misfolded glycoprotein carrying a CTS region for Golgi targeting and retention will be subjected to the same ERAD pathway like the luminal protein or the membrane‐anchored ER‐resident one. To answer this question, we examined the fate of MNS1‐SUBEX‐C57Y‐GFP where the CTS region of Arabidopsis Golgi‐α mannosidase I (MNS1) is attached to the folding‐defective SUBEX‐C57Y domain and GFP (Figure 2a). We transiently expressed MNS1‐SUBEX‐C57Y‐GFP in *N*. *benthamiana* leaf epidermal cells and analysed the protein expression by immunoblots. Substantial amounts of MNS1‐SUBEX‐C57Y‐GFP carrying the misfolded protein domain were only detectable in the presence of kifunensine (Figure 2b). By contrast, MNS1‐SUBEX‐GFP carrying the non‐mutated SUBEX domain fused to the CTS region of MNS1 accumulated also in the absence of the ERAD inhibitor and displayed only a slight increase in the presence of kifunensine. Upon transient expression of MNS1‐SUBEX‐C57Y‐GFP in *N*. *benthamiana* leaf epidermal cells, a very faint fluorescence signal resembling ER localisation was observed 48 h after infiltration without any substantial Golgi labelling (Figure 2c). MNS1‐SUBEX‐GFP, conversely, was found primarily in the Golgi apparatus and co‐localized with the Golgi marker *N*‐acetylglucosaminyltransferase I fused to mRFP (GnTI‐mRFP). These findings are consistent with the presence of a functional ER export and Golgi retention signal in the MNS1 CTS region (Liebminger *et al*., 2009). Therefore, the misfolded glycoprotein is subjected to ER retention, whereas the folding‐competent protein is efficiently targeted to the Golgi.

In stable transformed Arabidopsis wild‐type plants, MNS1‐SUBEX‐GFP was well expressed. By contrast, a clear dependency on mannose trimming was detected for MNS1‐SUBEX‐C57Y‐GFP as seen by the accumulation of the GFP‐tagged protein in kifunensine‐treated leaves from wild‐type (Figure 2d). Consistent with this finding, MNS1‐SUBEX‐C57Y‐GFP accumulated in the *mns45* double mutant, in *os9* and *sel1l* and the degradation was slower in *os9* when compared with Col‐0 (Figure 2e). Protein extracts from MNS1‐SUBEX‐C57Y‐GFP *os9* seedlings were subjected to Endo H or PNGase F digestion to investigate the glycosylation status. MNS1‐SUBEX‐C57Y‐GFP displayed a clear shift in mobility upon digestion with Endo H as well as with PNGase F indicating the presence of oligomannosidic *N*‐glycans that are typical for ER‐retained proteins (Figure 2f). MNS1‐SUBEX‐GFP, conversely, was resistant to Endo H in wild‐type *N*. *benthamiana* or Arabidopsis *os9* (Figure S1). To further corroborate this finding, we analysed transgenic *fut11 fut12* lines expressing MNS1‐SUBEX‐GFP. Glycoproteins that are modified in the Golgi in *fut11 fut12* carry complex *N*‐glycans lacking core α1,3‐fucose which are susceptible to digestion by PNGase F (Tretter *et al*., 1991; Strasser *et al*., 2004). The presence of Endo H‐resistant and PNGase F‐sensitive *N*‐glycans on MNS1‐SUBEX‐GFP is in full agreement with its predominant Golgi targeting and retention mediated by the MNS1‐CTS region (Figure 2f). This difference in subcellular localisation is also seen when *os9* seedlings expressing the two different constructs were analysed under the confocal microscope (Figure 2c).

Separation of membrane and soluble fractions showed that MNS1‐SUBEX‐C57Y‐GFP was concentrated in the membrane fraction in protein extracts from *os9* seedlings as already observed for GCSI‐SUBEX‐C57Y‐GFP (Figure 2g). Thus, our data demonstrate that the membrane‐anchored MNS1‐SUBEX‐C57Y‐GFP with a functional Golgi targeting and retention signal is efficiently retained in the ER and subjected to a glycan‐dependent ERAD pathway involving the ERAD factors MNS4/MNS5, OS9 and SEL1L.

Similarly, experiments with ST‐SUBEX‐C57Y‐GFP carrying the well‐known *trans*‐Golgi targeting sequence from rat α2,6‐sialyltransferase (Boevink *et al*., 1998; Saint‐Jore *et al*., 2002) resulted in ER retention and glycan‐dependent degradation (Figure S2). Taken together, these data highlight a dominant role of the ER quality control process over the ER exit and Golgi retention signals present in the CTS region of Golgi‐resident type II membrane proteins.

### A polytopic transmembrane variant of SUBEX‐C57Y carrying a functional Golgi targeting and retention signal is targeted to the vacuole

**Figure 3:**
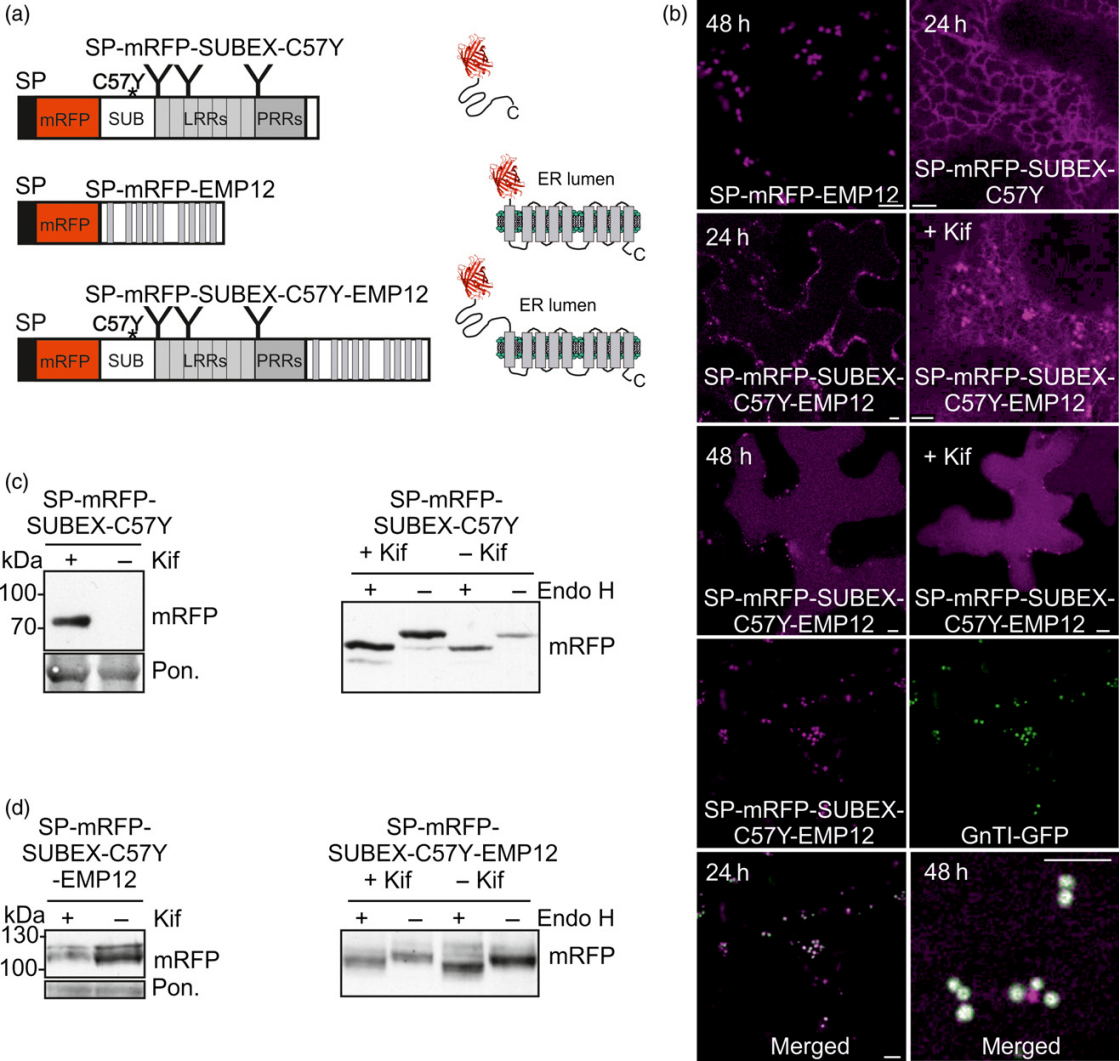
A misfolded Golgi retention signal‐containing multi‐pass transmembrane protein is routed to the vacuole in *N*. *benthamiana*.(a) Schematic illustration of SP‐mRFP‐SUBEX‐C57Y, SP‐mRFP‐EMP12 and SP‐mRFP‐SUBEX‐C57Y‐EMP12.(b) Confocal images of SP‐mRFP‐EMP12, SP‐mRFP‐SUBEX‐C57Y and SP‐mRFP‐SUBEX‐C57Y‐EMP12 transiently expressed for the indicated times in *N*. *benthamiana* leaves. Co‐expression with the Golgi marker GnTI‐GFP revealed co‐localization 24 h post infiltration and labelling of distinct puncta 48 h after infiltration (only the merged image is shown). Scale bars = 5 μm.(c) Immunoblot analysis (48 h after infiltration) and Endo H digestion of SP‐mRFP‐SUBEX‐C57Y transiently expressed in *N*. *benthamiana* leaves.(d) Immunoblot analysis and Endo H digestion of SP‐mRFP‐SUBEX‐C57Y‐EMP12 transiently expressed in *N*. *benthamiana* leaves (after 24 h). [Colour figure can be viewed at http://wileyonlinelibrary.com].

To analyse the fate of an ERAD substrate attached to a Golgi‐resident multi‐pass transmembrane domain protein, we fused SP‐mRFP‐SUBEX‐C57Y to the C‐terminal region of Arabidopsis ENDOMEMBRANE PROTEIN 12 (EMP12) (Figure 3a). Previously it was shown that EMP12 resides in the plant Golgi and the C‐terminal cytosolic region of EMP12 harbour efficient ER export as well as Golgi retention signals (Gao *et al*., 2012). To confirm the functionality of the targeting region we fused SP‐mRFP to the nine transmembrane domain containing C‐terminal region of EMP12 and expressed the chimeric SP‐mRFP‐EMP12 protein transiently in *N*. *benthamiana* leaves. As expected, SP‐mRFP‐EMP12 labelled puncta resembling the Golgi (Figure 3b). Furthermore, to demonstrate that SUBEX‐C57Y is still subjected to ERAD when fused to SP‐mRFP, we expressed SP‐mRFP‐SUBEX‐C57Y in *N*. *benthamiana*. Consistent with the fate of a glycoprotein ERAD substrate, SP‐mRFP‐SUBEX‐C57Y is located in the ER (Figure 3b), degraded in a glycan‐dependent manner and carries Endo H sensitive *N*‐glycans (Figure 3c).

Transiently expressed SP‐mRFP‐SUBEX‐C57Y‐EMP12 displayed ER and Golgi‐like structures 24 h after infiltration and predominately vacuolar localisation 48 h after infiltration. In addition to the vacuolar staining, SP‐mRFP‐SUBEX‐C57Y‐EMP12 labelled smaller vesicular structures when expressed in *N*. *benthamiana* leaves. To identify these cellular structures we performed co‐localisation with GnTI‐GFP. While some of the puncta displayed co‐localisation with the Golgi marker, additional fluorescent structures were present (Figure 3b). By contrast, SP‐mRFP‐EMP12 displayed a very good co‐localisation with GnTI‐GFP without labelling of additional puncta (Figure S3). In the presence of kifunensine the localisation of SP‐mRFP‐SUBEX‐C57Y‐EMP12 was unaffected and the protein levels were not increased when transiently expressed (Figure 3b, d). Up to four bands corresponding to the expected molecular weight of approximately 110 kDa were detected for SP‐mRFP‐SUBEX‐C57Y‐EMP12 by SDS‐PAGE and immunoblotting. Upon digestion with Endo H, the major band shifted indicating the presence of substantial amounts of protein carrying oligomannosidic *N*‐glycans. However, a minor fraction of SP‐mRFP‐SUBEX‐C57Y‐EMP12 was insensitive to endoglycosidase digestion even in the presence of kifunensine when all *N*‐glycans should be Endo H sensitive suggesting that this SP‐mRFP‐SUBEX‐C57Y‐EMP12 fraction is not accessible for deglycosylation or present in an unglycosylated form (Figure 3d).

**Figure 4:**
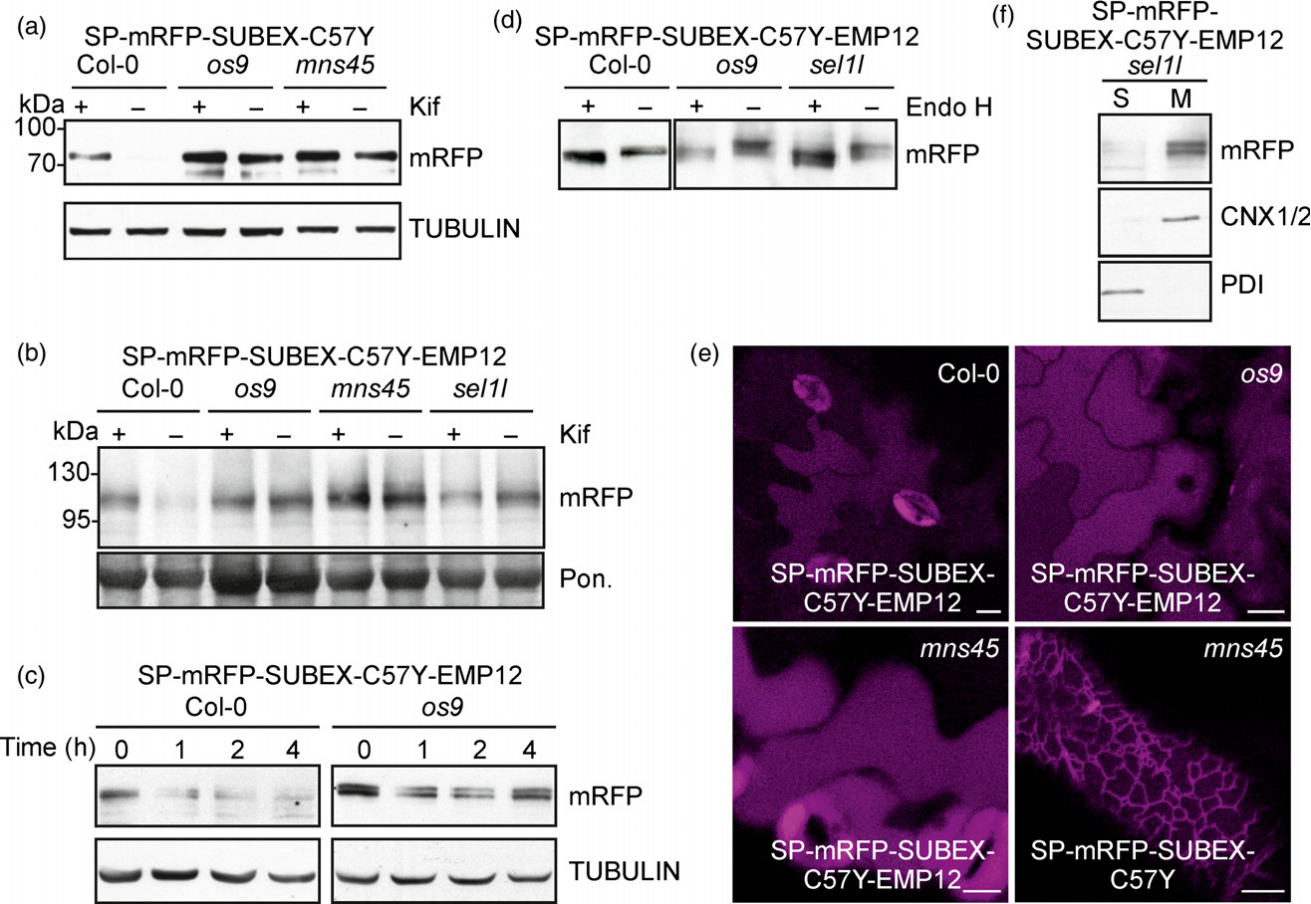
A misfolded Golgi retention signal‐containing multi‐pass transmembrane protein is partially degraded by the glycan‐dependent ERAD pathway in Arabidopsis.(a) Immunoblot analysis of transgenic Col‐0, *os9* and *mns45* expressing SP‐mRFP‐SUBEX‐C57Y.(b) Immunoblot analysis of transgenic Col‐0, *os9*,* mns45* and *sel1l* expressing SP‐mRFP‐SUBEX‐C57Y‐EMP12. The full blot is shown in Figure S4.(c) Degradation of SP‐mRFP‐SUBEX‐C57Y‐EMP12 in the presence of 100 μg ml^−1^ cycloheximide (CHX). The experiment was done four times with similar results. An additional repetition is shown in Figure S5.(d) Endo H digestion of SP‐mRFP‐SUBEX‐C57Y‐EMP12.(e) Confocal images of Arabidopsis expressing SP‐mRFP‐SUBEX‐C57Y‐EMP12 and SP‐mRFP‐SUBEX‐C57Y. Scale bars = 10 μm.(f) SP‐mRFP‐SUBEX‐C57Y‐EMP12 distribution in soluble (S) and membrane fractions (M). [Colour figure can be viewed at http://wileyonlinelibrary.com].

To investigate the fate of the polytopic membrane‐anchored protein more in detail, we generated transgenic Arabidopsis expressing SP‐mRFP‐SUBEX‐C57Y, SP‐mRFP‐EMP12 and SP‐mRFP‐SUBEX‐C57Y‐EMP12. In wild‐type, SP‐mRFP‐SUBEX‐C57Y was subjected to degradation and the protein was stabilized in ERAD mutants like *os9* (Figure 4a). SP‐mRFP‐EMP12, on the other hand, was not degraded in wild‐type and mutant lines (Figure S3). SP‐mRFP‐SUBEX‐C57Y‐EMP12 expression was hardly detectable by immunoblots in the different Arabidopsis lines. We treated 36 independent transformed Col‐0 wild‐type plants with the ERAD inhibitor kifunensine and found that the majority of them displayed an accumulation of the expected band at 110 kDa in the presence of kifunensine. For the other lines the expression levels were too low to observe such an effect. In contrast with wild‐type, kifunensine did not further increase SP‐mRFP‐SUBEX‐C57Y‐EMP12 levels in *os9*,* mns45* and *sel1l* (Figures 4b and S4). CHX‐treatment repeatedly showed that the degradation is faster in Col‐0 compared to *os9* (Figures 4c and S5) indicating that SP‐mRFP‐SUBEX‐C57Y‐EMP12 is an unstable protein in wild‐type plants. Consistent with the results from the transient expression in *N*. *benthamiana*, most *N*‐glycans from SP‐mRFP‐SUBEX‐C57Y‐EMP12 were sensitive to Endo H (Figure 4d). Moreover, Col‐0, *os9* and *mns45* expressing SP‐mRFP‐SUBEX‐C57Y‐EMP12 displayed predominately vacuolar localisation (Figure 4e) and SP‐mRFP‐SUBEX‐C57Y‐EMP12 was mainly found in the membrane fraction (Figure 4f). Taken together, these results suggest that multiple mechanisms are involved in the disposal of SP‐mRFP‐SUBEX‐C57Y‐EMP12 in Arabidopsis as we observed a contribution from the HRD1 machinery as well as trafficking from the ER to the vacuole.

### An ER‐retained EMP12 variant of SUBEX‐C57Y is degraded in a glycan‐dependent manner without trafficking to the vacuole

The differences observed for the degradation of SP‐mRFP‐SUBEX‐C57Y‐EMP12 and other SUBEX‐C57Y‐containing ERAD substrates may arise from the presence of the nine transmembrane domain region which provides either a structural hindrance for efficient glycan‐dependent ERAD or contains a dominant ER export and Golgi retention signal. To distinguish between these possibilities we expressed SP‐mRFP‐SUBEX‐C57Y‐TMD9, harbouring only the last transmembrane domain and the C‐terminal region required for ER export and Golgi retention from EMP12 (Gao *et al*., 2012). As a control we attached the same EMP12 region to SP‐mRFP (SP‐mRFP‐TMD9). When transiently expressed in *N*. *benthamiana* leaves, we found that both SP‐mRFP‐TMD9 and SP‐mRFP‐SUBEX‐C57Y‐TMD9 were present in the ER without any Golgi localization (Figure S6). This finding is unexpected because SP‐mRFP‐TMD9 lacks any misfolded protein domain or sequence motif for ER retention and suggests that additional domains from EMP12 are required for ER exit and Golgi retention. Comparison of protein extracts from infiltrated *N*. *benthamiana* leaves or stable transformed Arabidopsis showed that SP‐mRFP‐SUBEX‐C57Y‐TMD9 is stabilized in the presence of kifunensine indicating that this C‐terminally membrane‐anchored protein is subjected to glycan‐dependent ERAD similar to the previously analysed folding‐defective proteins.

**Figure 5:**
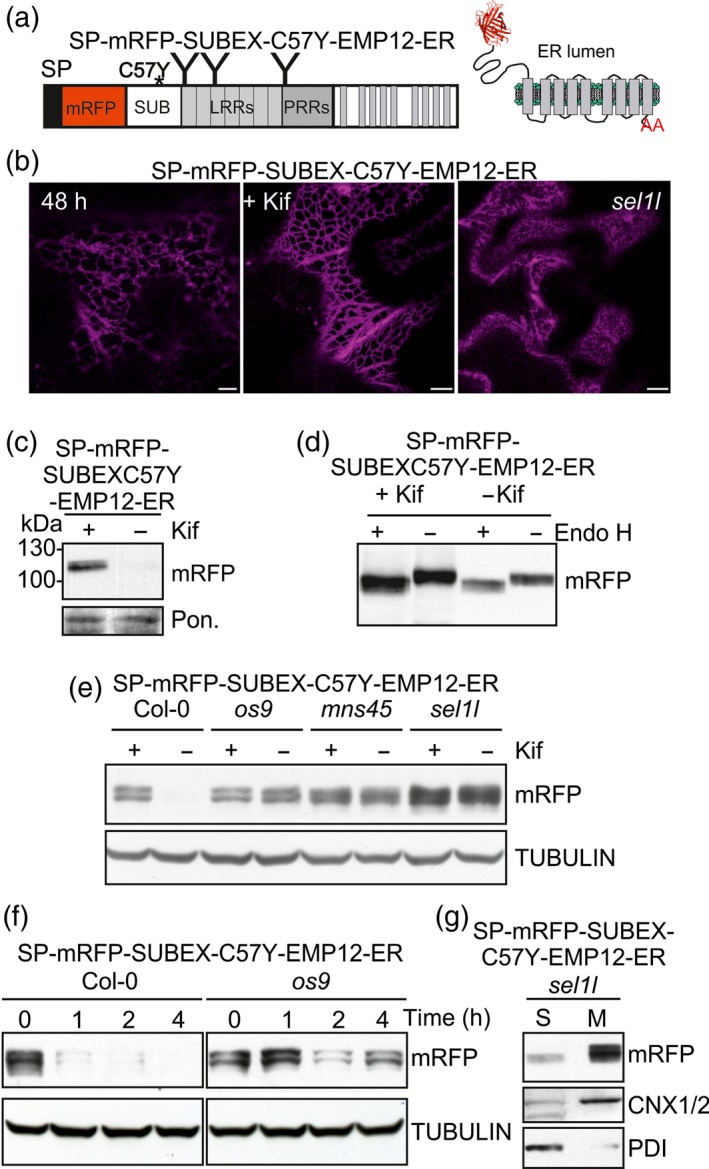
ER‐retained SP‐mRFP‐SUBEX‐C57Y‐EMP12 is degraded by the glycan‐dependent ERAD pathway.(a) Schematic illustration of SP‐mRFP‐SUBEX‐C57Y‐EMP12‐ER.(b) Confocal images of SP‐mRFP‐SUBEX‐C57Y‐ER transiently expressed in *N*. *benthamiana* leaves. Confocal image of Arabidopsis *sel1l* expressing SP‐mRFP‐SUBEX‐C57Y‐EMP12‐ER. Scale bars = 10 μm.(c) Immunoblot analysis of kifunensine (Kif) treated *N*. *benthamiana* transiently expressing SP‐mRFP‐SUBEX‐C57Y‐EMP12‐ER. Protein extracts were analysed 24 h after infiltration.(d) Endo H digestion of transiently expressed SP‐mRFP‐SUBEX‐C57Y‐EMP12‐ER.(e) Immunoblot analysis of transgenic Col‐0, *os9*,* mns45* and *sel1l* expressing SP‐mRFP‐SUBEX‐C57Y‐EMP12‐ER. The full blot is shown in Figure S4.(f) Degradation of SP‐mRFP‐SUBEX‐C57Y‐EMP12‐ER in the presence of 100 μg ml^−1^ cycloheximide (CHX). The experiment was done two times with similar results.(g) SP‐mRFP‐SUBEX‐C57Y‐EMP12‐ER distribution in soluble (S) and membrane fractions (M). [Colour figure can be viewed at http://wileyonlinelibrary.com].

Next, we mutated two amino acid residues in the cytoplasmic tail of SP‐mRFP‐SUBEX‐C57Y‐EMP12 by substitution of valine at position 579 with alanine (V579A) and tyrosine at position 583 with alanine (Y583A) (Figure 5a). According to a previously published study, proteins carrying such a mutated EMP12 C‐terminal motif will result in ER accumulation (Gao *et al*., 2012). Indeed, when SP‐mRFP‐EMP12‐ER was expressed in *N*. *benthamiana*, ER localisation was observed without any labelling of Golgi bodies (Figure S7). In contrast to SP‐mRFP‐SUBEX‐C57Y‐EMP12, SP‐mRFP‐SUBEX‐C57Y‐EMP12‐ER labelled the ER (Figure 5b) and treatment with kifunensine caused accumulation of the protein when transiently expressed (Figure 5c). Endo H digestion of protein extracts led to a shift in mobility without the appearance of Endo H‐resistant bands (Figure 5d). Consistent with these findings, SP‐mRFP‐SUBEX‐C57Y‐EMP12‐ER was subjected to glycan‐dependent degradation in Arabidopsis wild‐type, stabilized in *os9*,* mns45* and *sel1l* (Figures 5e and S4) and displayed ER localization (Figure 5b). SP‐mRFP‐SUBEX‐C57Y‐EMP12‐ER was much faster cleared in wild‐type compared to *os9* when protein synthesis was blocked and found predominately in the membrane fraction (Figure 5f, g). In contrast to SP‐mRFP‐SUBEX‐C57Y‐EMP12, these data indicate that the same variant lacking a functional ER export motif is exclusively targeted to the glycan‐dependent ERAD pathway.

### A misfolded BRI1 domain fused to EMP12 is not subjected to glycan‐dependent ERAD

**Figure 6:**
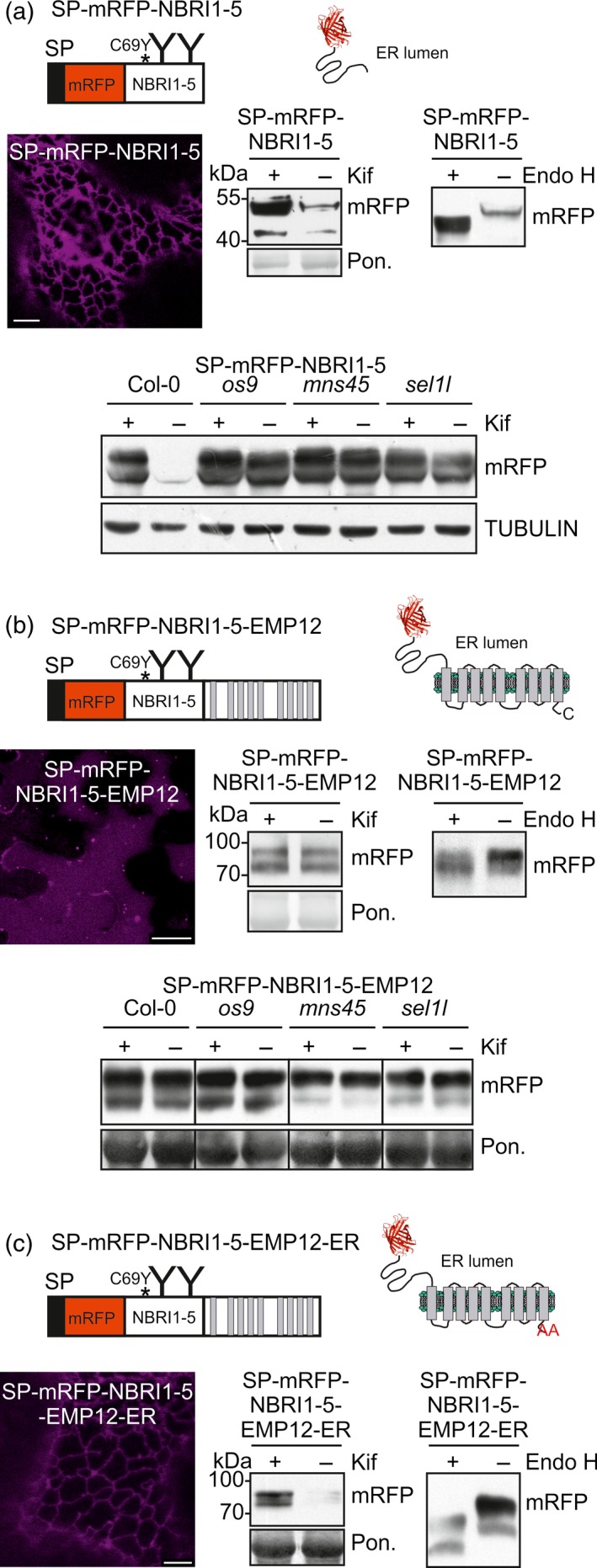
Misfolded SP‐mRFP‐NBRI1‐5‐EMP12 is not subjected to glycan‐dependent ERAD.(a) Schematic illustration of SP‐mRFP‐NBRI1‐5. The asterisk denotes the position of the amino acid change (C69Y) leading to misfolding; ‘Y’ indicates the position of the *N*‐glycosylation sites. NBRI1‐5 corresponds to amino acids 24–212 from BRI1 carrying *N*‐glycosylation sites Asn112 and Asn154. Confocal image (48 h after infiltration) (scale bar = 5 μm), immunoblot analysis (24 h after infiltration) and Endo H digestion of SP‐mRFP‐NBRI1‐5 transiently expressed in *N*. *benthamiana* leaves. Immunoblot analysis of transgenic Col‐0, *os9*,* mns45* and *sel1l* expressing SP‐mRFP‐NBRI1‐5.(b) Schematic illustration of SP‐mRFP‐NBRI1‐5‐EMP12. Confocal image (48 h after infiltration) (scale bar = 25 μm), immunoblot analysis (30 h after infiltration) and Endo H digestion of SP‐mRFP‐NBRI1‐5‐EMP12 transiently expressed in *N*. *benthamiana* leaves. Immunoblot analysis of transgenic Col‐0, *os9*,* mns45* and *sel1l* expressing SP‐mRFP‐NBRI1‐5‐EMP12.(c) Schematic illustration of SP‐mRFP‐NBRI1‐5‐EMP12‐ER. Confocal image (48 h after infiltration) (scale bar = 5 μm), immunoblot analysis (48 h after infiltration) and Endo H digestion of SP‐mRFP‐NBRI1‐5‐EMP12‐ER transiently expressed in *N*. *benthamiana* leaves. [Colour figure can be viewed at http://wileyonlinelibrary.com].

The predominant vacuolar location of SP‐mRFP‐SUBEX‐C57Y‐EMP12 suggests that the C‐terminal transmembrane domain containing EMP12 region with the functional ER export and Golgi retention signal is dominant over ER quality control processes. To examine whether the EMP12 region has a similar effect on another misfolded glycoprotein, we expressed chimeric proteins consisting of an *N*‐terminal domain from BRI1‐5 (NBRI1‐5) fused to SP‐mRFP with or without the EMP12 region. NBRI1‐5 carries the C69Y amino acid substitution that leads to misfolding and ER retention of BRI1 (Hong *et al*., 2008) and two *N*‐glycosylation sites (Figure 6a). SP‐mRFP‐NBRI1‐5 was located in the ER, carried Endo H sensitive *N*‐glycans and was subjected to glycan‐dependent degradation in *N*. *benthamiana* and Arabidopsis. Consequently, we concluded that SP‐mRFP‐NBRI1‐5 behaves like a typical glycan‐dependent ERAD substrate. By contrast, SP‐mRFP‐NBRI1‐5‐EMP12 highlighted mainly the vacuole and displayed no apparent glycan‐dependent degradation (Figure 6b) indicating that the EMP12 ER export region is dominant over ER quality control for this misfolded protein. By contrast, a variant with the mutated EMP12 ER export motif, SP‐mRFP‐NBRI1‐5‐EMP12‐ER, was subjected to glycan‐dependent ERAD (Figure 6c).

Taken together, our data show that an ER‐retained aberrant glycoprotein fused to the nine transmembrane domain regions from EMP12 is subjected to MNS4/5‐OS9‐SEL1L‐HRD1‐mediated ERAD similar to other integral membrane proteins or soluble ERAD substrates. The presence of the EMP12‐domain with the intact ER export and Golgi retention signal causes targeting of misfolded glycoproteins from the ER to the vacuole.

## Discussion

**Figure 7:**
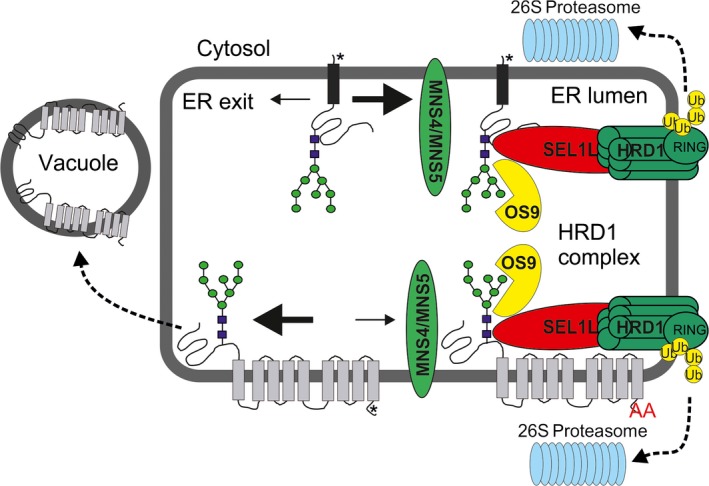
Working model illustrating the fate of different misfolded integral membrane proteins. Misfolded glycoproteins carrying a single transmembrane domain and functional ER export signal (marked by an asterisk) from Golgi‐resident type II membrane proteins like MNS1 or ST are efficiently retained in the ER and degraded by glycan‐dependent ERAD involving the HRD1 complex. Binding of the misfolded protein to the SEL1L‐HRD1 complex may lead to retro‐translocation into the cytosol, ubiquitination (stimulated by the RING domain of HRD1) and degradation by the 26S proteasome. ER‐retained variants of misfolded glycoprotein carrying a polytopic membrane domain (such as the C‐terminal region from EMP12 with nine transmembrane domains; ‘AA’ in the C‐terminal cytosolic part indicates the mutated ER export motif) are also efficiently cleared by glycan‐dependent ERAD showing that the HRD1 complex can handle degradation of various substrate types. If a misfolded glycoprotein carries a polytopic transmembrane domain with a strong functional Golgi targeting motif (such as in EMP12, marked by an asterisk), then the ERAD substrate is mainly targeted to an alternative degradation route (indicated by the bold arrow) involving trafficking to the vacuole. Please note that the downstream events of ERAD involving retro‐translocation, ubiquitination and proteasomal degradation are not very well established in plants. [Colour figure can be viewed at http://wileyonlinelibrary.com].

The role of membrane anchoring domains in the quality control of terminally misfolded proteins is poorly understood in plants. Here, we investigated the contribution of different ER/Golgi targeting and retention regions for degradation of a misfolded variant of the glycosylated extracellular domain from Arabidopsis STRUBBELIG. Remarkably, we found that the misfolded glycoproteins were efficiently retained in the ER and targeted to glycan‐dependent ERAD when ER export and Golgi retention signals from Golgi‐resident type II membrane proteins were present (Figure 7). This finding shows that ER quality control is quite stringent and dominant over the signals present in the CTS regions of these proteins. Golgi‐resident type II membrane proteins from mammals or plants have typically rather short cytoplasmic tails which carry several basic amino acids involved in anterograde trafficking from the ER to the Golgi apparatus (Giraudo and Maccioni, 2003; Schoberer *et al*., 2009; Schoberer and Strasser, 2011). Mutation of all three basic amino acids in the 11 amino acid long cytoplasmic tail of tobacco GnTI led to ER retention of this *cis*/medial Golgi‐resident glycosyltransferase. Confocal microscopy studies in the presence of brefeldin A or upon co‐expression of a GTP‐locked version of the small GTPase SAR1 provide evidence that such Golgi enzymes are transported by COPII carriers (Schoberer *et al*., 2009). Presumably, the interaction with the cytoplasmic tail recruits SAR1 and COPII coat components like SEC23/SEC24 to ER exit sites. Our data suggest that ER quality control precedes this event and prevents the packaging into ER‐to‐Golgi carriers. Our data are fully consistent with previous studies showing that ER quality control is dominant over signals for the vacuolar sorting of soluble proteins (Foresti *et al*., 2003), signals for tonoplast sorting (Maîtrejean *et al*., 2011) and domains that promote the formation of ER‐located protein bodies (de Virgilio *et al*., 2008).

Misfolded proteins harboring a single C‐terminal transmembrane domain (SP‐mRFP‐SUBEX‐C57Y‐TMD9 or the nine transmembrane domains from EMP12 are all degraded by the HRD1 complex in a glycan‐dependent manner when retained in the ER. These data indicate that the presence of different membrane anchoring regions *per se* does not interfere with ERAD and does not sort the misfolded proteins to different disposal routes. In yeast and mammals, hallmarks of ERAD are retro‐translocation of the aberrant protein from the ER to the cytoplasm and degradation by the 26S proteasome (Vembar and Brodsky, 2008). Whether these steps are essential for glycan‐dependent ERAD in plants remains to be shown. In plants, retro‐translocation of a non‐glycosylated GFP fusion protein and glycosylated plant toxins have been described (Brandizzi *et al*., 2003; Marshall *et al*., 2008). For the plant toxins, however, the clearance was independent of mannose trimming indicating the involvement of an alternative glycan‐independent pathway.

For the EMP12 variant with the intact ER export and Golgi retention motif, we observed vacuolar localization suggesting targeting to a different disposal pathway. However, we do not have proof that intact SP‐mRFP‐SUBEX‐C57Y‐EMP12 reaches the vacuole. On immunoblots considerable amounts of free mRFP were detected (Figure S4), which explain the detectable fluorescence in the lumen of the vacuole. We do not know where exactly the cleavage takes place, but the presence of very low amounts of free mRFP for the ER‐retained SP‐mRFP‐SUBEX‐C57Y‐EMP12‐ER variant, suggest that the cleavage from the rest of the protein takes place in a post‐ER compartment like the PVC or vacuole. A similar scenario has been discussed previously for a membrane‐anchored invertase targeted to the vacuole (Barrieu and Chrispeels, 1999).

In stable transformed Arabidopsis, however, we could still observe an involvement of ERAD factors. Based on our results, we propose that ER quality control and ER export processes compete for the substrate and a pool of the misfolded protein stays in the ER where it is degraded by the classic ERAD pathway like other analyzed ERAD substrates (Figure 7). When ER retention by ER quality control processes is less dominant as suggested by the results from SP‐mRFP‐NBRI1‐5‐EMP12, no noticeable ERAD activity takes place and the protein is transported predominately to the vacuole. If, however, ER retention is increased, for example, by the mutation of the EMP12 ER export signal, then the aberrant protein is subjected to glycan‐dependent ERAD. In summary, we propose that ER quality control and ER export are in constant competition and protein intrinsic determinants such as the nature of the misfolded domain (SUBEX‐C57Y or NBRI1‐5) and the type of ER export signal (EMP12 or MNS1/ST) determine the fate of the misfolded protein. A similar scenario has been previously described for ligands of the major ER chaperone BiP, which is subjected to vacuolar degradation as an alternative to ERAD (Pimpl *et al*., 2006) and for an aberrant maize storage protein (Foresti *et al*., 2008). In line with different fates of membrane‐anchored non‐native proteins, recent studies from mammals and yeast provide evidence that misfolded GPI‐anchored proteins are poor ERAD substrates and instead sorted primarily to the lysosome/vacuole for degradation (Satpute‐Krishnan *et al*., 2014; Sikorska *et al*., 2016).

In mammals, different ERAD factors are required for the degradation of aberrant soluble and integrated membrane proteins (Bernasconi *et al*., 2010). Members of the mammalian OS9 lectin family which deliver soluble glycosylated clients to the membrane‐embedded HRD1 complex, are dispensable for ERAD of membrane proteins. Interestingly, we found no involvement of distinct ERAD factors for degradation of soluble or membrane proteins. All tested substrates were dependent on the same set of ERAD factors: (i) MNS4/MNS5, which initially trims the *N*‐glycan to generate the glycan signal for degradation (Hüttner *et al*., 2014b); (ii) OS9, which is supposed to bind to the MNS4/MNS5‐processed oligomannosidic *N*‐glycan (Hüttner *et al*., 2012; Su *et al*., 2012); and (iii) SEL1L, which interacts with OS9 as well as with HRD1 and binds to misfolded regions of the substrate (Liu *et al*., 2011; Su *et al*., 2011). It is still unclear if HRD1‐dependent ERAD in plants requires ubiquitination of ERAD substrates such as SUBEX‐C57Y or BRI1‐5 variants. Efforts to detect ubiquitination of these ERAD substrates have so far been unsuccessful, but for the glycan‐dependent ERAD substrate BRI1‐9 ubiquitination involving the HRD1 interacting protein EBS7 has been shown (Liu *et al*., 2015). Alternatively, the HRD1 ubiquitin ligase domain may be involved in autoubiquitination of HRD1 or ubiquitination of other ERAD factors. In yeast, autoubiquitination of lysine residues in the cytosolic RING domain of HRD1 is important for retro‐translocation of ERAD substrates (Baldridge and Rapoport, 2016). Interestingly, ubiquitination by Arabidopsis HRD1A/B regulates the protein levels of the E2 ubiquitin conjugating enzyme UBC32 (Chen *et al*., 2016) and UBC32 negatively controls the stability of OS9 (Chen *et al*., 2017). Whether UBC32, which is a proposed component of the DOA10 ERAD complex, participates directly or indirectly via OS9 in degradation of topologically divers ERAD substrates, remains to be shown.

Although, misfolded proteins fused to the EMP12 region with the strong ER export and Golgi retention signal predominately evade ER quality control and ER retention, little accumulates in the Golgi. Either the presence of the misfolded domain leads to vacuolar trafficking with a considerable amount of the protein bypassing the Golgi (De Marchis *et al*., 2013), or there is an additional quality control check in the Golgi that detects non‐native luminal domains and targets aberrant proteins to degradation instead of Golgi retention (Arvan *et al*., 2002). The absence of Golgi‐processed complex *N*‐glycans is in favor for direct trafficking to the vacuole as described previously for other ER‐resident proteins (Tamura *et al*., 2004). The involvement of a Golgi quality control process detecting misassembled transmembrane domains has been suggested recently for mammalian cells (Briant *et al*., 2017). A vacuolar trafficking route, similar to the one observed for the EMP12‐containing ERAD substrates has been described for EMP12‐GFP which carries the GFP at the C‐terminal end and thus interferes with Golgi retention mediated by COPI subunits (Gao *et al*., 2012). The COPI interaction takes place on the cytoplasmic side of the Golgi membrane and involves binding to conserved amino acids within EMP proteins (Woo *et al*., 2015). Recognition of a misfolded luminal polypeptide domain requires an interaction in the Golgi lumen without any direct involvement of cytoplasmic regions. Consequently, the selection for Golgi‐to‐vacuole trafficking of misfolded glycoprotein appears distinct from the mistargeting due to the presence of GFP at the C‐terminal end or absence of Golgi retention signals. How the misfolded protein is recognized and targeted to such distinct Golgi‐independent/dependent trafficking routes is currently unclear.

In summary, we found that the presence of single or multiple transmembrane domains fused to aberrant glycoproteins does not obstruct HRD1 complex‐mediated glycan‐dependent ERAD. However, if a strong ER export signal is present such as in EMP12, the balance between ER quality control and ER exit is considerably shifted to ER exit leading to trafficking to the vacuole. Identification of key factors and determinants involved in disposal of non‐native proteins is essential to understand mechanisms that maintain cellular protein homeostasis.

## Experimental procedures

### Plant material

Arabidopsis plants were grown under long‐day conditions (16 h light/8 h dark) at 22°C. T‐DNA insertion lines *os9*,* sel1l* and the *mns45* mutants were described recently (Hüttner *et al*., 2012, 2014b). Transgenic Arabidopsis plants were generated by floral dipping and subsequent selection of seedlings on hygromycin‐containing 0.5 ×  Murashige and Skoog (MS) medium. For treatment with kifunensine, cut leaves from 4–5‐week‐old soil grown plants were incubated for 24 h in 0.5 ×  MS medium supplemented with 1% sucrose and 50 μM kifunensine (Santa Cruz Biotechnology). For all transformed constructs at least 12 independent Arabidopsis plants were initially analyzed. In the subsequent generations, kifunensine treatment was repeated for selected independent lines. Treatment with other inhibitors was done with 12‐day‐old Arabidopsis seedlings in the same way by adding 100 μg/ml cycloheximide (Sigma‐Aldrich, Vienna, Austria). *N*. *benthamiana* plants were grown on soil under long‐day conditions at 25°C. For inhibitor treatments in *N*. *benthamiana*, 20 μM kifunensine were infiltrated into leaves together with agrobacteria carrying plasmids for transient expression of proteins.

### Plasmid construction

To generate p47‐SUBEX‐C57Y for expression of SP‐SUBEX‐C57Y‐GFP, the DNA encoding a mutant variant of the extracellular domain from Arabidopsis STRUBBELIG (AT1G11130, amino acids 1–341, Cys at position 57 substituted by Tyr) was excised from p20‐SUBEX‐C57Y by *Xba*I/*Bam*HI digestion and cloned into expression vector p47 (Hüttner *et al*., 2014b). In p47, GFP fusion proteins are expressed under the control of the Arabidopsis ubiquitin 10 gene promoter (Grefen *et al*., 2010). To generate the CTS region containing SUBEX‐C57Y expression vectors, SUBEX‐C57Y was amplified from p20‐SUBEX‐C57Y with primers SUB_17F/_16R (Table S1), digested with *Bam*HI/*Bgl*II and cloned into the *Bam*HI site of p47. The GCSI‐CTS region (Schoberer *et al*., 2009) was subsequently cloned into the *Xba*I/*Bam*HI site to generate p47‐GCSI‐SUBEX‐C57Y. p47‐MNS1‐SUBEX‐C57Y and p47‐ST‐SUBEX‐C57Y were generated by insertion of the MNS1‐CTS (Liebminger *et al*., 2009) and ST‐CTS (Boevink *et al*., 1998) region, respectively. p47‐MNS1‐SUBEX was made using the same cloning strategy with PCR amplification from p20‐SUBEX (Hüttner *et al*., 2014a). p117 was obtained by insertion of a synthetic DNA fragment (GeneArt gene synthesis, Thermo Fisher Scientific) carrying the SP sequence from Arabidopsis calnexin 1 (CNX1) fused to mRFP into the *Xba*I/*Sal*I site of p47. To generate p117‐SP‐mRFP‐SUBEX‐C57Y, the PCR product obtained by amplification with SUB_17F/_16R was inserted into the *Bam*HI site of p117. To generate p111 for the expression of Arabidopsis EMP12‐fusion proteins (AT1G10950, carrying amino acids 220 to 589 of EMP12) with the functional ER export and Golgi retention motif (Gao *et al*., 2012), the EMP12 coding region was amplified from Arabidopsis cDNA with EMP12_6F/_7R, digested with *Bam*HI/*Xho*I and cloned into *Bam*HI/*Sal*I digested p47. To generate p111‐mRFP and p111‐mRFP‐SUBEX‐C57Y, the PCR fragments SP‐mRFP (amplified with CNX1_12F/mRFP21‐R) and SP‐mRFP‐SUBEX‐C57Y (amplified with CNX1_12F/SUB_16R), respectively, were digested with *Spe*I/*Bgl*II and cloned into *Xba*I/*Bam*HI site of p111. The p112 variants for expression of the ER‐retained EMP12‐fusion protein (amino acids 220 to 589, amino acids Val579 and Tyr583 mutated to Ala) were generated in the same way by insertion of a PCR product amplified from Arabidopsis cDNA with EMP12_6F/_10R. To generate p113 for expression of the variants fused to the C‐terminal region of EMP12 which comprises the last transmembrane domain and the cytoplasmic tail (amino acids 544 to 589), the corresponding EMP12 region was amplified from cDNA with EMP12_11F/_7R and cloned into p47. The SP‐mRFP and SP‐mRFP‐SUBEX‐C57Y fragments were cloned as described for p111. To generate p117‐NBRI1‐5, the DNA region coding for amino acids 24–212 from Arabidopsis BRI1 (AT4G39400) was amplified from cDNA with primers BRI1_25F/_27R, *Spe*I/*Bam*HI digested and cloned into the *Xba*I/*Bam*HI site of p117‐mRFP. For p111‐mRFP‐NBRI1‐5 and p112‐mRFP‐NBRI1‐5, the PCR product obtained with BRI1_25F/_26R was *Spe*I digested and cloned into the *Xba*I site of p111‐mRFP and p112, respectively.

### Confocal microscopy

For subcellular localization studies, the agrobacterium strain UIA143 carrying one of the different ERAD substrate expression constructs was diluted to an OD_600_ of 0.2 and expressed in leaf epidermal cells of 5‐week‐old *N*. *benthamiana* plants following agrobacterial leaf infiltration as described previously (Schoberer *et al*., 2009). Organelle markers and other co‐expressed proteins were co‐infiltrated with an OD_600_ of 0.05–0.1. Sections of infiltrated leaves were analyzed 24 h and 48 h after infiltration on a Leica TCS SP5 confocal microscope as described in detail previously (Schoberer *et al*., 2014).

### Immunoblotting

Protein extraction and digestion with endoglycosidase H (Endo H) and peptide‐*N*‐glycosidase F (PNGase F) (both from New England Biolabs) were carried out as described recently (Hüttner *et al*., 2014a). Primary antibodies against GFP (Miltenyi Biotec), RFP (Chromotek), HA (Roche), CALNEXIN (Agrisera), ACTIN (Agrisera) and TUBULIN (Sigma‐Aldrich) were commercially available. The protein disulfide isomerase (PDI) antibody was generated in our group and described in detail previously (Farid *et al*., 2011). To isolate membrane and soluble fractions, 200 mg seedlings were extracted with 400 μl extraction buffer (100 mm Tris‐HCl, pH 7.5, 25% (w/w) sucrose, 5% (v/v) glycerol, 10 mm EDTA, 1 mm DTE). Cell debris was removed by centrifugation at 600 ***g*** for 3 min. The supernatant was centrifuged at 16 000 ***g*** for 1 h. The pellet (M fraction) was extracted in SDS‐PAGE loading buffer and subjected together with the supernatant (S fraction) to SDS‐PAGE and immunoblotting.

## Conflict of interest

The authors declare no conflict of interest.

## Supporting information


**Figure S1.** Endo H and PNGase F digestion of MNS1‐SUBEX‐GFP.
**Figure S2.** The ERAD pathway is dominant over Golgi targeting/retention signals from rat α2,6‐sialyltransferase (ST).
**Figure S3.** Golgi localization of SP‐mRFP‐EMP12.
**Figure S4.** Detection of free mRFP on immunoblots.
**Figure S5.** Degradation of SP‐mRFP‐SUBEX‐C57Y‐EMP12 in the presence of cycloheximide (CHX).
**Figure S6.** SP‐mRFP‐SUBEX‐C57Y‐TMD9 is retained in the ER and subjected to glycan‐dependent ERAD.
**Figure S7.** Subcellular localisation of SP‐mRFP‐EMP12‐ER harbouring mutations in the ER export motif.

**Table S1.** List of used primers.
