# Supplementary material for: The glycan‐dependent ERAD machinery degrades topologically diverse misfolded proteins
Source: Plant J. 2018 Mar 14;94(2):246–59. doi: 10.1111/tpj.13851 (PMC5900737; doi:10.1111/tpj.13851)
Supplement: Supplementary file 1 — Figure S1. Endo H and PNGase F digestion of MNS1‐SUBEX‐GFP. Figure S2. The ERAD pathway is dominant over Golgi targeting/retention signals from rat α2,6‐sialyltransferase (ST). Figure S3. Golgi localization of SP‐mRFP‐EMP12. Figure S4. Detection of free mRFP on immunoblots. Figure S5. Degradation of SP‐mRFP‐SUBEX‐C57Y‐EMP12 in the presence of cycloheximide (CHX). Figure S6. SP‐mRFP‐SUBEX‐C57Y‐TMD9 is retained in the ER and subjected to glycan‐dependent ERAD. Figure S7. Subcellular localisation of SP‐mRFP‐EMP12‐ER harbouring mutations in the ER export motif. [file TPJ-94-246-s001.pdf]

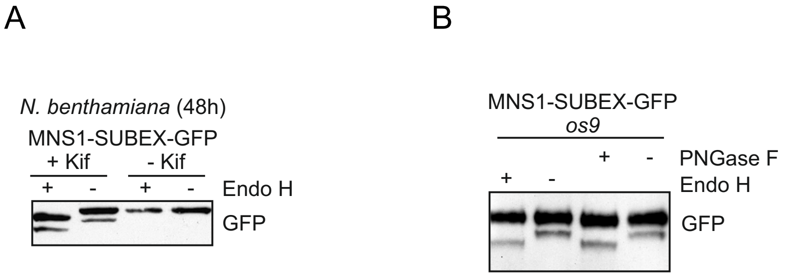


**Figure S1. Endo H and PNGase F digestion of MNS1-SUBEX-GFP.**
**(A)** MNS1-SUBEX-GFP was transiently expressed in *N. benthamiana* in the presence or absence of kifunensine (Kif) and subjected to Endo H digestion. (**B**) MNS1-SUBEX-GFP expressed in *Arabidopsis os9* was subjected to Endo H and PNGase F digestion.

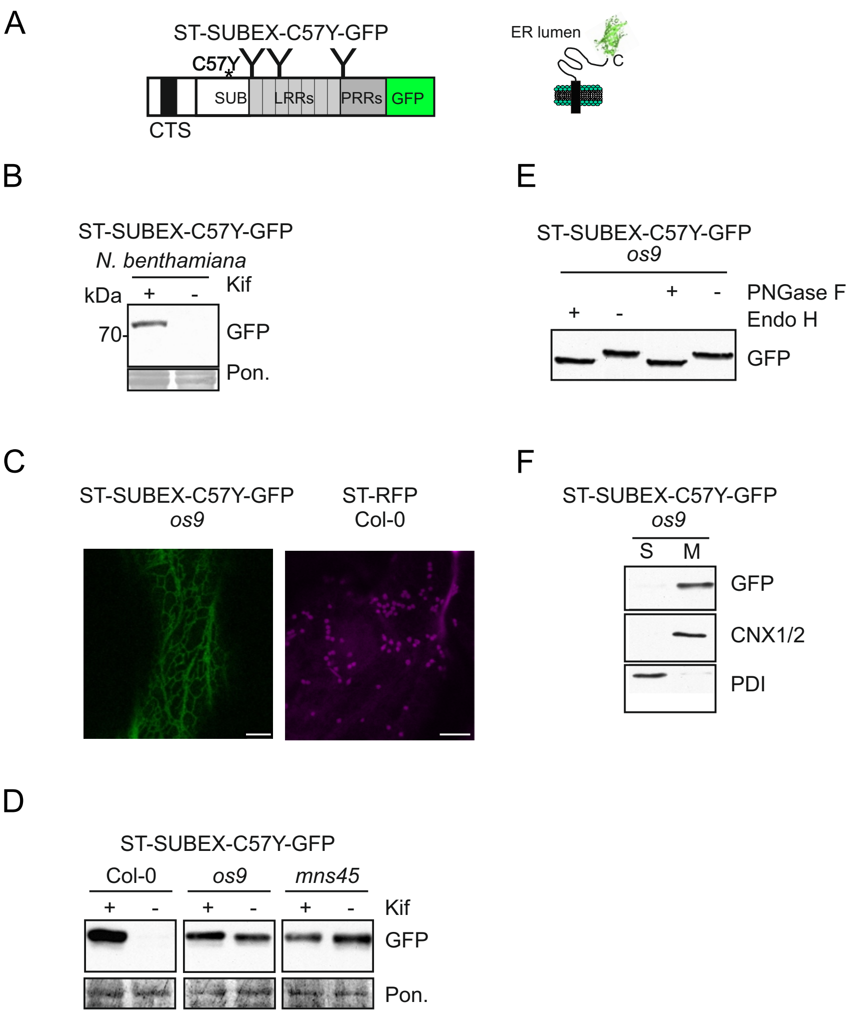


**Figure S2. The ERAD pathway is dominant over Golgi targeting/retention signals from rat α2,6-sialyltransferase (ST).**

(**A**) Schematic illustration of ST-SUBEX-C57Y-GFP. The signal peptide from SP-SUBEX-C57Y-GFP is replaced by the ST-CTS region. (**B**) Immunoblot of ST-SUBEX-C57Y-GFP transiently expressed in *N. benthamiana* leaves in the presence or absence of 20 µM kifunensine (Kif). Proteins were analysed 48 h after infiltration. (**C**) Confocal images of 12-day-old *Arabidopsis* seedlings expressing ST-SUBEX-C57Y-GFP. Scale bars = 7.5 µm. (**D**) Immunoblot analysis of protein extracts from 4-5-week-old *Arabidopsis* expressing ST-SUBEX-C57Y-GFP. (**E**) Endo H and PNGase F digestion of ST-SUBEX-C57Y-GFP from 4-5-week-old *Arabidopsis*. (**F**) Detection of ST-SUBEX-C57Y-GFP in soluble (S) and membrane fractions (M) of 12-day-old *os9* seedlings.

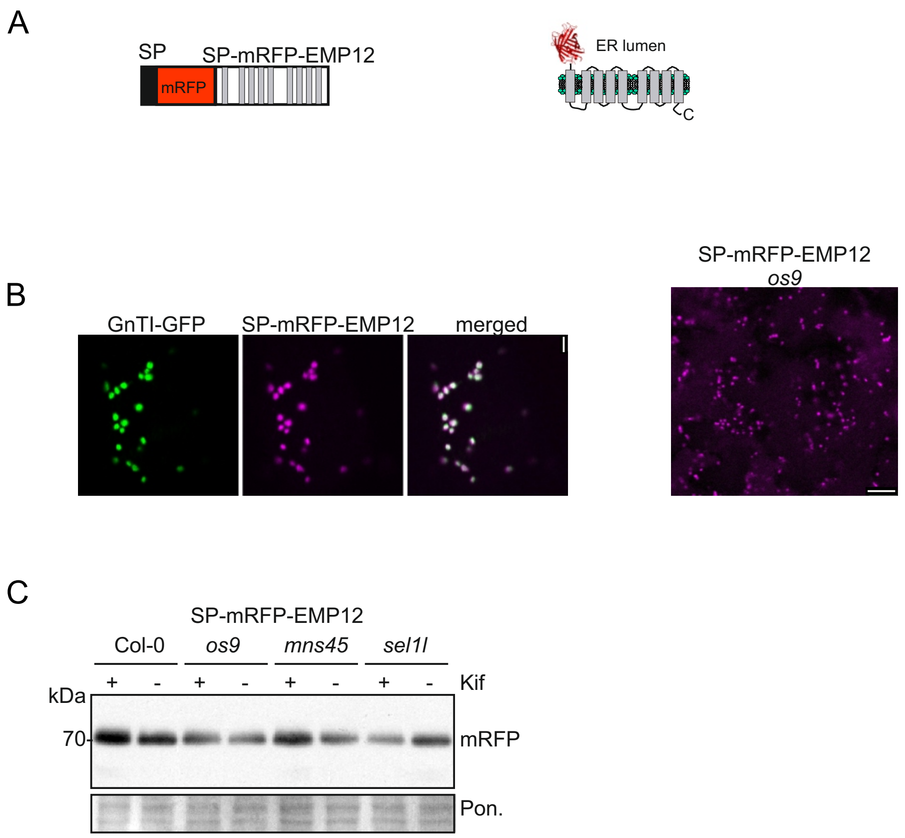


**Figure S3. Golgi localization of SP-mRFP-EMP12.**

(**A**) Schematic illustration of SP-mRFP-EMP12. The C-terminal part of *Arabidopsis* EMP12 carrying nine transmembrane domains is fused to SP-mRFP. (**B**) Confocal images of SP-mRFP-EMP12 (48 h after infiltration) transiently expressed in *N. benthamiana* leaves (scale bar = 25 µm) as well as of *Arabidopsis os9* leaves expressing SP-mRFP-EMP12 (scale bar = 10 µm). (**C**) Immunoblot analysis of protein extracts from 4-5-week-old *Arabidopsis* expressing SP-mRFP-EMP12.

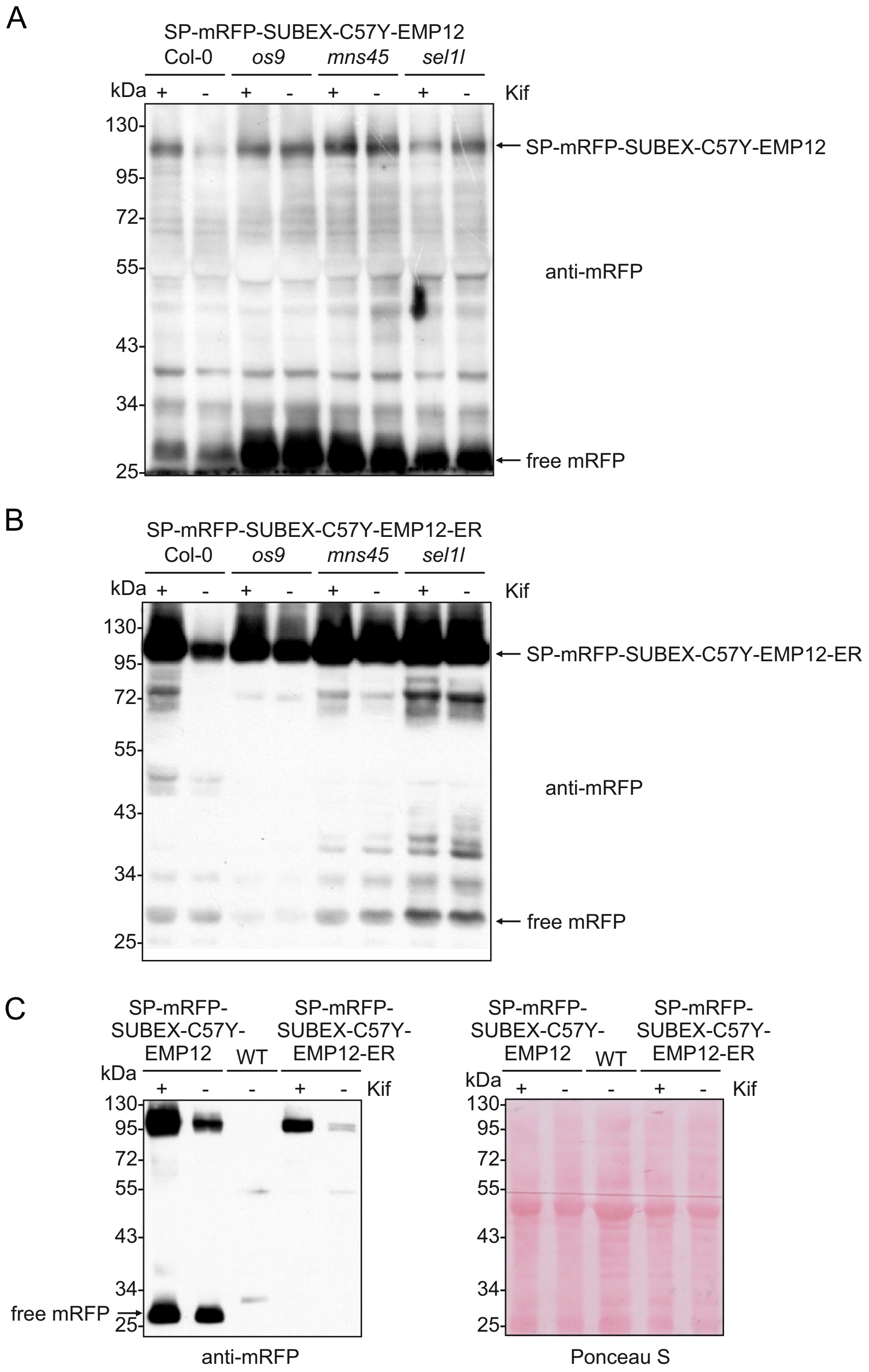


**Figure S4. Detection of free mRFP on immunoblots.**

(**A**) Immunoblot analysis of transgenic Col-0, *os9*, *mns45* and *sel1l* expressing SP-mRFP-SUBEX-C57Y-EMP12. The presence of cleaved free mRFP is indicated. The image corresponds to the one shown in Figure 4b and displays the molecular weight range from 25-130 kDa. (**B**) Immunoblot analysis of transgenic Col-0, *os9*, *mns45* and *sel1l* expressing SP-mRFP-SUBEX-C57Y-EMP12-ER. The presence of cleaved free mRFP is indicated. The image corresponds to the one shown in Figure 5e and displays the molecular weight range from 25-130 kDa. (**C**) Comparison of Col-0 expressing SP-mRFP-SUBEX-C57Y-EMP12 and Col-0 expressing SP-mRFP-SUBEX-C57Y-EMP12-ER on the same blot. A protein extract from a non-transformed Col-0 WT plant is included as a control.

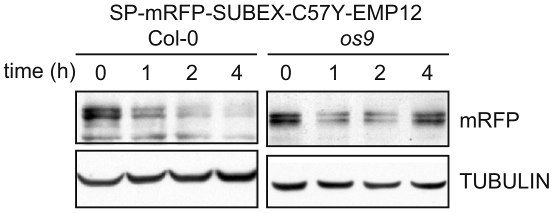


**Figure S5. Degradation of SP-mRFP-SUBEX-C57Y-EMP12 in the presence of cycloheximide (CHX).**

12-day-old *Arabidopsis* wild-type and os9 seedlings expressing SP-mRFP-SUBEX-C57Y-EMP12 were incubated for the indicated time with 100 µg/ml CHX. Proteins were extracted and subjected to SDS-PAGE followed by immunoblotting with antibodies against RFP and tubulin.

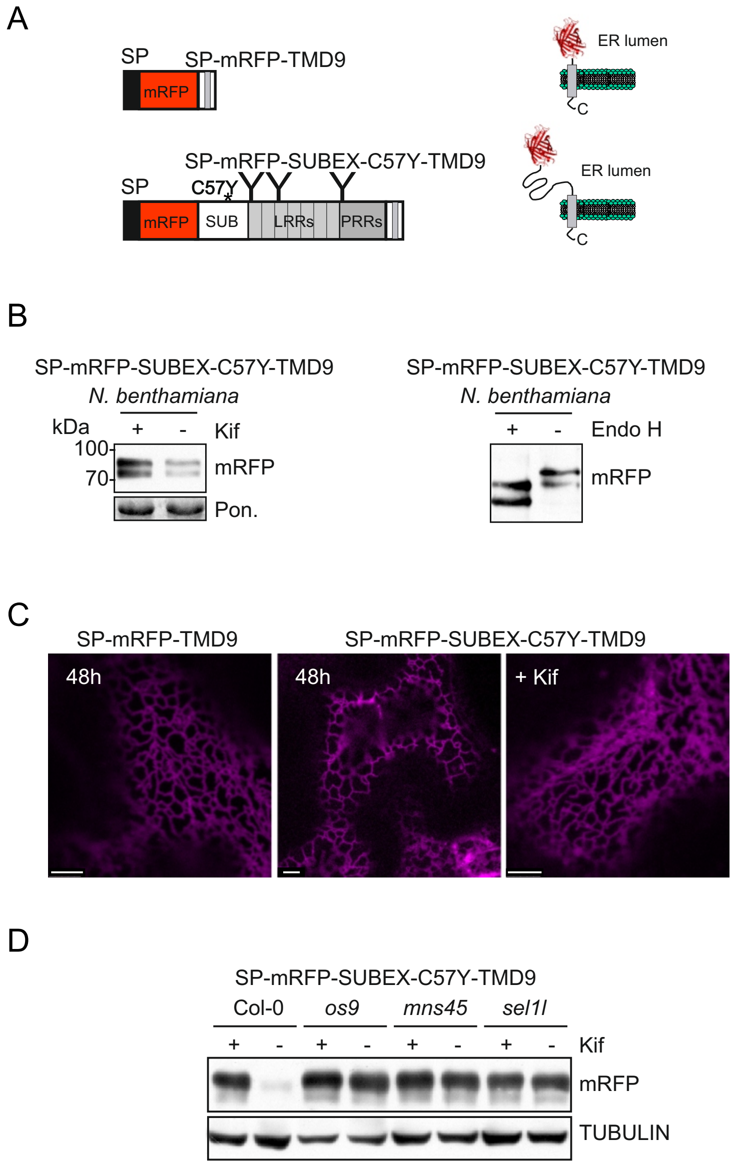


**Figure S6. SP-mRFP-SUBEX-C57Y-TMD9 is retained in the ER and subjected to glycan-dependent ERAD.**
(**A**) Schematic illustration of SP-mRFP-TMD9 and SP-mRFP-SUBEX-C57Y-TMD9. These chimeric proteins contain the C-terminal region of *Arabidopsis* EMP12 consisting of the last transmembrane domain and the C-terminal Golgi targeting and retention signal that faces the cytosol. (**B**) Immunoblot analysis of SP-mRFP-SUBEX-C57Y-TMD9 transiently expressed in *N. benthamiana* leaves in the presence or absence of 20 µM kifunensine (Kif) and after Endo H digestion. Proteins were analysed 48 h after infiltration. (**C**) Confocal images of SP-mRFP-TMD9 and SP-mRFP-SUBEX-C57Y-TMD9 (48 h after infiltration) transiently expressed in *N. benthamiana* leaves. Scale bars = 5 µm. (**D**) Immunoblot analysis of protein extracts from 4-5-week-old *Arabidopsis* expressing SP-mRFP-SUBEX-C57Y-TMD9.

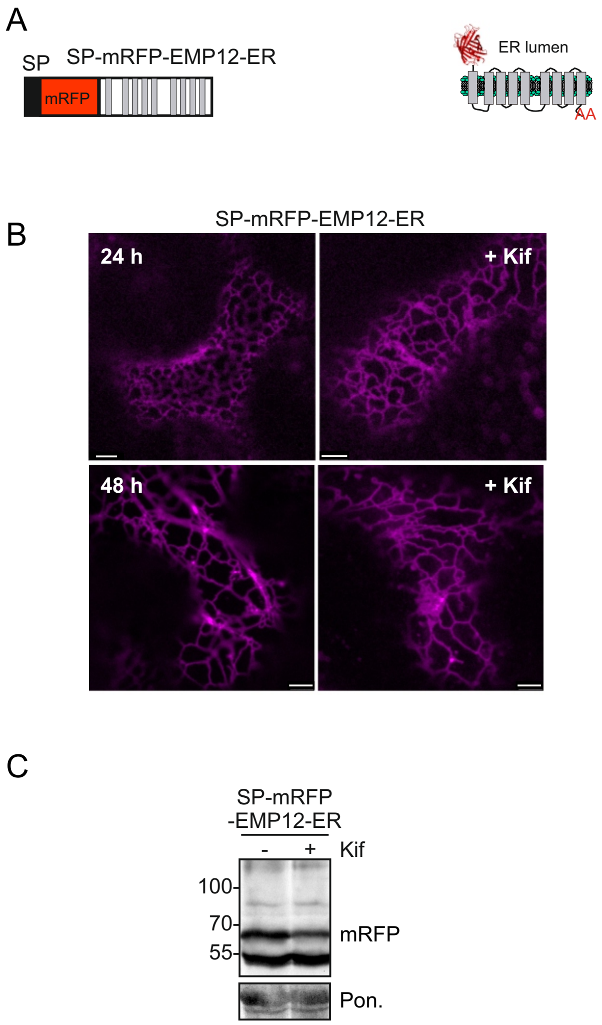


**Figure S7. Subcellular localisation of SP-mRFP-EMP12-ER harbouring mutations in the ER export motif.**

(**A**) Schematic illustration of SP-mRFP-EMP12-ER. The cytoplasmic C-terminal region carries two amino acid substitutions that inactivate the ER export signal of *Arabidopsis* EMP12. (**B**) Confocal images of SP-mRFP-EMP12-ER (24 and 48 h after infiltration) transiently expressed in *N. benthamiana* leaves. Scale bars = 5 µm. (**C**) Immunoblot analysis of SP-mRFP-EMP12-ER transiently expressed in *N. benthamiana* leaves in the presence or absence of 20 µM kifunensine (Kif) (48 h after infiltration).
