# Supplementary material for: The glycan‐dependent ERAD machinery degrades topologically diverse misfolded proteins
Source: Plant J. 2018 Mar 14;94(2):246–59. doi: 10.1111/tpj.13851 (PMC5900737; doi:10.1111/tpj.13851)
Supplement: Supplementary file 2 — Table S1. List of used primers. [file TPJ-94-246-s002.pdf]

Table S1: List of used primers.

Name	Sequence (5’-3’)
BRI1_25F	tataACTAGTTTTCAAGCTTCACCATCTCAGTCT
BRI1_26R	tataACTAGTGATTTTGTTTCCGCTAATCGCTAA
BRI1_27R	tataGGATCCTCAGATTTTGTTTCCGCTAATCGCTA
CNX1_12F	tataACTAGTATGAGACAACGGCAACTATTTTCC
EMP12_6F	tataGGATCCATCCACTGGTTCTCCATCTTTAAC
EMP12_7R	tataCTCGAGCTAGTCGCACTTGATGTTTCTGTAG
EMP12_10R	tataCTCGAGCTAGTCGCACTTGATGTTTCTGGCGATCCTCCTTGCAAACAGATTTG
EMP12_11F	tataGGATCCGGATTCTTCCAGACAAGCTTC
mRFP21-R	tataAGATCTAGCTCTAGAAGCACCAGTAGAATG
SUB_16R	tataAGATCTTCTTTGAGTGGACCAGAATTTTCC
SUB_17F	tataGGATCCGTTACTAATCTACGAGATGTTTCG
